# Prognostic significance of the stress hyperglycemia ratio and admission blood glucose in diabetic and nondiabetic patients with spontaneous intracerebral hemorrhage

**DOI:** 10.1186/s13098-024-01293-0

**Published:** 2024-03-04

**Authors:** Shengru Liang, Xiaoxi Tian, Fei Gao, Minghao Man, Qi Wang, Jianwei Li, Lihong Li, Yang Yang

**Affiliations:** 1 Department of Endocrinology, Tangdu Hospital, Fourth Military Medical University, 710038 Xi’an, China; 2 Department of Emergency, Tangdu Hospital, Fourth Military Medical University, 710038 Xi’an, China; 3 Department of Neurosurgery, Tangdu Hospital, Fourth Military Medical University, 710038 Xi’an, China

**Keywords:** Intracerebral hemorrhage, Admission blood glucose, Stress-induced hyperglycemia ratio, Diabetic status, Poor outcome

## Abstract

**Background:**

The role of stress hyperglycemia ratio (SHR) on the prognosis of spontaneous intracerebral hemorrhage (ICH) in patients with different diabetic status has not been elucidated. This study aimed to evaluate the prognostic value of SHR and admission blood glucose (ABG) for the short- and long-term mortality in diabetic and nondiabetic populations with ICH.

**Method:**

Participants with ICH were retrospectively retrieved from the Medical Information Mart for Intensive Care (MIMIC-IV). The primary outcome was all-cause 30-day and 1-year mortality. The association of SHR and ABG with the primary outcomes in diabetic and nondiabetic cohorts were assessed by Cox proportional hazard regression.

**Results:**

Overall, 1029 patients with a median age of 71.09 (IQR: 60.05–81.97) were included. Among them, 548 (53%) individuals were male, and 95 (19%) as well as 323 (31%) ones experienced the 30-day and 1-year mortality, respectively. After adjusting for confounding variables, individuals in quintile 5 of SHR had significantly higher risk of the 30-day and 1-year mortality than those in quintile 1 in the whole cohort (30-day mortality: HR 3.33, 95%CI 2.01–5.51; 1-year mortality: HR 2.09, 95% CI 1.46-3.00) and in nondiabetic patients (30-day mortality: HR 4.55, 95%CI 2.33–8.88; 1-year mortality: HR 3.06, 95%CI 1.93–4.86), but no significant difference was observed in diabetic patients. Similar results were observed for ABG as a categorical variable. As continuous variable, SHR was independently correlated with the 30-day and 1-year mortality in both of the diabetic and nondiabetic cohorts (30-day mortality: HR 2.63, 95%CI 1.50–4.60. 1-year mortality: HR 2.12, 95%CI 1.33–3.39), but this correlation was only observed in nondiabetic cohort for ABG (HR 1.00, 95%CI 0.99–1.01 for both of the 30-day and 1-year mortality). Moreover, compared with ABG, SHR can better improve the C-statistics of the original models regarding the 30-day and 1-year outcomes, especially in patients with diabetes (*p* < 0.001 in all models).

**Conclusion:**

SHR might be a more useful and reliable marker than ABG for prognostic prediction and risk stratification in critically ill patients with ICH, especially in those with diabetes.

**Supplementary Information:**

The online version contains supplementary material available at 10.1186/s13098-024-01293-0.

## Introduction

Spontaneous, nontraumatic, intracerebral hemorrhage (ICH) accounts for about 10–20% of all types of strokes which contributes the highest morbidity and mortality [1]. Despite much efforts made in the past decades, there are still few therapeutic options to improve prognosis after ICH [2]. Therefore, identifying modifiable factors that influence the outcome of ICH is important for that it may provide new targets for treatment.

Hyperglycemia at admission has been reported to be associated with poor outcomes in patients admitted with ICH [3–5]. The American Stroke Association guidelines also state that serum glucose should be monitored and hyperglycemia should be avoided in patients with ICH (Class I; Level of Evidence C) [6]. However, the admission blood glucose (ABG) level cannot entirely reflect the acute hyperglycemic state, which is also affected by the chronic glucose level. Therefore, to identify acute hyperglycemia more accurately, the stress hyperglycemia ratio (SHR) that combined ABG and glycosylated hemoglobin A1c (representing glycemic status of the past 2 ~ 3 months) was proposed. Since then, investigators have verified the predictive value of SHR on the prognosis of ACS and AMI [7–10]. More recently, the association between SHR and the higher risk of poor outcomes in patients with ICH was also reported [11, 12]. Nevertheless, as previous studies revealed that ABG and hyperglycemia was positively associated with short- and long-term poor outcomes in non-diabetic, but not diabetic individuals with ICH [13, 14], it is essential to clarify whether the impact of SHR on the prognosis of ICH is also dependent on different status of glucose metabolism.

In this study, we aimed to compare the predictive value of two measures of hyperglycemia (SHR and ABG) on the short- and long-term all-cause mortality in critically ill patients with ICH and in subgroups divided by diabetes mellitus, which may be useful to stratify and recognize individuals at high risk of all-cause mortality for timely healthcare management.

## Method

### Data sources

We conducted a retrospective study by retrieving data from the Medical Information Mart for Intensive Care (MIMIC-IV version 2.2) database [15]. MIMIC-IV is consisted of de-identified health-related data of more than 190,000 patients admitting to the intensive critical care units of the Beth Israel Deaconess Medical Center between 2008 and 2019. Since MIMIC-IV is de-identified to remove patients’ information and our study is centered around the analysis of this accessible database, which had already received the approval of an institutional review board (IRB), the requirement for patient informed consent and ethics approval is not indispensable.

### Study population and data extraction

PostgresSQL (version 13.7.2) and Navicate Premium (version 16) was used to run structure query language (SQL) and extract data from MIMIC-IV. One author (Yang Yang) received proper authorization to access the database (Record ID: 48,776,647), and was responsible for data extraction. We included adult patients (> 18 years of age) with a diagnosis of ICH according to the International Classification of Diseases, 9th and 10th Revision (ICD9 and ICD10). The exclusion criteria were as follows: patients with ICH (1) dying or leaving within 24 h since intensive care unit (ICU) admission; (2) with multiple ICU admissions, for whom only the data of the first admission due to ICH were extracted; (3) with missing ABG and HbA1c data on the first day of admission. Finally, a total of 1028 patients were extracted and enrolled in this study.

For the final cohort, we collected the following data: (1) age and gender; (2) comorbidity as coded and defined by the ICD-9 and ICD-10 (Additional file 1: Table S1); (3) severity of illness scores on the first day of ICU admission, which include acute physiology score III (APSIII), simplified acute physiological score II (SAPSII), sequential organ failure assessment (SOFA), and oxford acute severity of illness score (OASIS); (4) the first result of glucose and HbA1c since ICU admission; (5) the average laboratory results on the first day of ICU admission; (6) the mean values of vital signs during the first 24 h of ICU stay; (7) medication data during ICU stay including usage of vasoactive drugs, renal replacement treatment (RRT) and invasive ventilation.

**Fig. 1:**
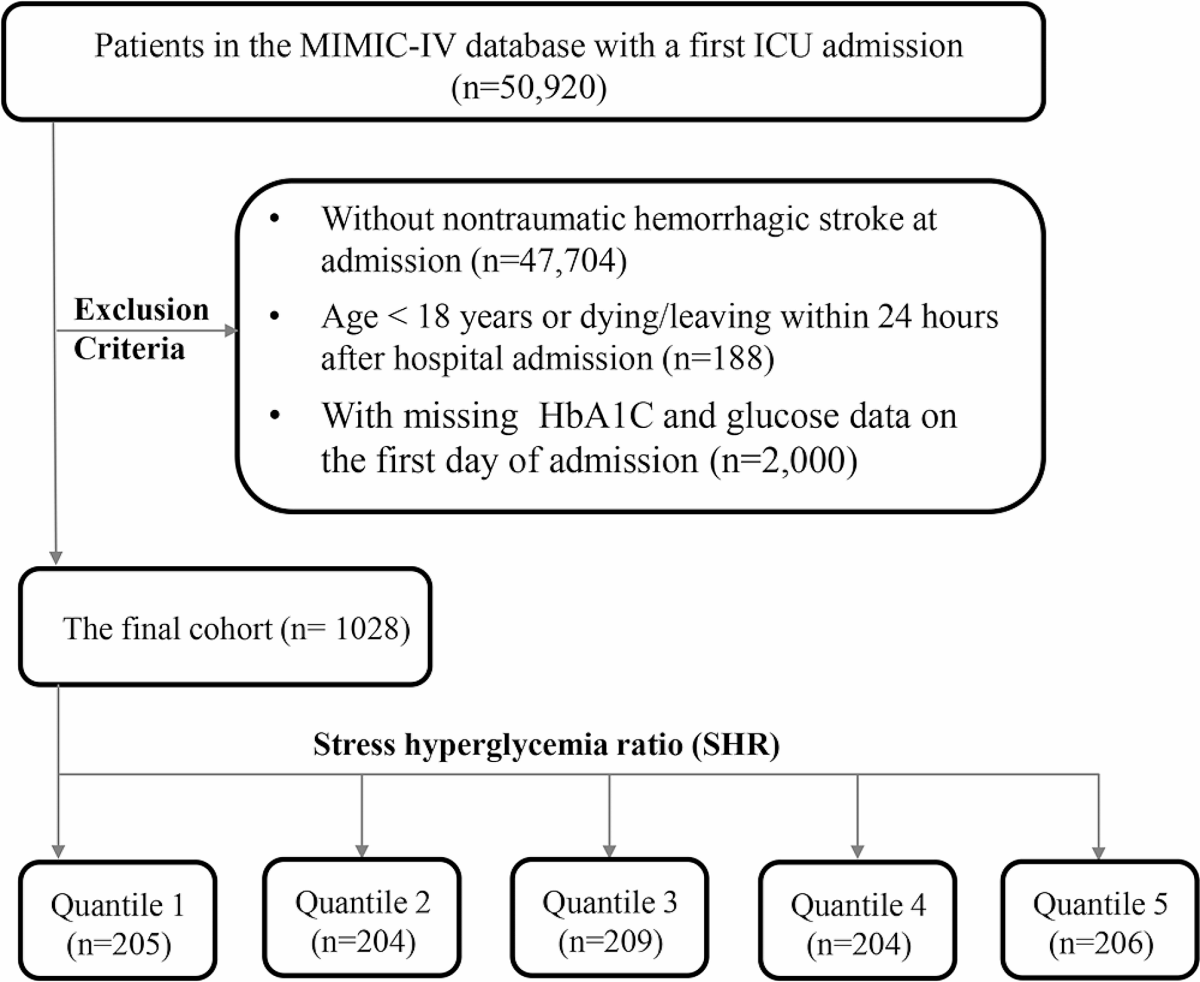
Flowchart illustrating the screening process of critically ill patients with ICH from the MIMIC-IV database

Diabetes was diagnosed according to the definition of the American Diabetes Association as (1) a definite history of diabetes, or (2) HbA1c value ≥ 6.5% [16]. The value of SHR was calculated as follows: SHR = ABG(mg/dL)/(28.7 × HbA1c(%) − 46.7) [17]. Then, the final cohort was divided into five groups according to the quintile of the SHR. (Fig. 1).

### Clinical outcomes

The primary outcomes were all-cause 30-day and 1-year mortality. It is worth noting that the MIMIC-IV database restricts access to follow-up dates beyond one year from the last hospital discharge. Therefore, no patient was lost to follow-up within 1 year, but the database does not facilitate insights into patient mortality beyond the 1-year timeframe.

### Statistical analysis

The Shapiro-Wilk test was used to assess the distribution of continuous variables. Data were expressed as mean ± standard deviation (SD) for normally distributed variables, median (interquartile ranges) for non-normal data, and number (percentages) for categorical data. Normally distributed variables were compared by unpaired Student’ t test or ANOVA, and variables with a skewed distribution were compared by Mann-Whitney U-test or Kruskal-Wallis test. The chi-squared test or Fisher’s exact test were performed to assess the differences in categorial variables between groups.

Kaplan-Meier’s survival analysis combined with Log-rank test was then conducted to visualize and compare the probability of the 30-day and 1-year death among groups divided by quintile of the SHR and ABG. Before Cox regression analysis was conducted, the association of SHR and ABG with other continuous variables were assessed by Pearson’s correlation test or Spearman’s rank correlation test (Additional file 2: Figure S1). Univariable and multivariable Cox proportional hazard regression was then performed to evaluate the impact of SHR and ABG on the 30-day and 1-year outcomes. In multivariable analysis, we adjusted the variables related to the 30-day or 1-year mortality in univariate Cox regression with a *p* value < 0.01. Further, we used a restricted cubic spline regression model with four knots to evaluate the nonlinear association of SHR and ABG with the primary outcomes. Then, Harrell’s C-statistic was calculated to assess whether adding SHR or ABG into a model of the severity of illness scores (APSIII, SAPSII, SOFA, and OASIS) could improve the discrimination of the model for the 30-day and 1-year mortality. Finally, subgroup analyses were conducted by stratifying patients according to their diabetic status.

We use R software (version 4.3.1) for data analysis. “VIM” package of R was used to visualize the distribution of missing values. All of the collected variables had missing ratio less than 20% (Additional file 3: Figure S2). The “mice” package of R was adopted to address missing values by multiple imputation to obtain 5 imputation datasets in the process of Cox regression. Besides, the “corrplot” package was used to visualize the associations of SHR, ABG and HbA1c with continuous variables. “survminer” packages was used to conduct Kaplan-Meier’s survival analysis. “survivalROC” packages was used to estimate the cut-off values. A two-tailed *p* value of < 0.05 was considered statistically significant.

## Results

### Baseline characteristics

The median age of the whole cohort was 71.09 (IQR: 60.05–81.97) years and among them, 548 (53%) patients were male and 304 (30%) ones had diabetes. The 30-day and 1-year mortality of the study cohort were 19% and 31%, respectively. Besides, an ascending gradient with respect to the severity of illness scores, prevalence of diabetes, values of mean heart rate and respiratory rate, number of lung infection, ICU and hospital stay time, as well as the 30-day and 1-year mortality, whereas a descending gradient regarding value of mean arterial pressure was found from quintile 1 to quintile 5 of SHR (Table 1). Furthermore, patients with diabetes had higher score of APSIII, higher prevalence of congestive heart failure, renal diseases and hypertension, lower value of white blood cell count, and higher levels of ABG and HbA1c. While no difference was observed for the other variables between the two groups (Additional file 4: Table S2).

**Table 1:** Baseline characteristics of participants grouped by quintile of SHR ^a,b^

Variables	Total (*n* = 1028)	Q1 (*n* = 205)	Q2 (*n* = 204)	Q3 (*n* = 209)	Q4 (*n* = 204)	Q5 (*n* = 206)	*p*-value	
Age, years	71.09 (60.05, 81.97)	72.1 (62.44, 82.39)	72.96 (62.34, 82.28)	72.69 (63.21, 84.18)	67.81 (56.2, 79.31)	68.73 (56.89, 77.79)	< 0.001	
Male, n%	548 (53)	108 (53)	113 (55)	120 (57)	114 (56)	93 (45)	0.097	
GCS	14 (12, 15)	14 (13, 15)	14 (12, 15)	14 (11, 15)	14 (12, 15)	14 (11, 15)	0.332	
**Severe Score**								
APSIII	34 (25, 44)	32 (24, 43)	30 (21.75, 40)	34 (26, 44)	34 (26, 45)	40.5 (31, 51)	< 0.001	
SAPSII	31 (25, 39)	31 (25, 40)	30 (24, 37)	30 (24, 36)	31 (24, 38)	35 (28, 41)	< 0.001	
SOFA	3 (1, 4)	2 (1, 4)	2 (1, 4)	2 (1, 4)	3 (1, 4)	4 (2, 5)	< 0.001	
OASIS	30 (26, 36)	29 (25, 35)	29 (24, 34)	30 (26, 35)	30 (26, 35)	33 (28, 38)	< 0.001	
**Comorbidities, n(%)**								
MI	95 (9)	19 (9)	22 (11)	18 (9)	10 (5)	26 (13)	0.089	
CHF	157 (15)	36 (18)	33 (16)	30 (14)	25 (12)	33 (16)	0.623	
Diabetes	304 (30)	72 (35)	48 (24)	52 (25)	44 (22)	88 (43)	< 0.001	
Renal disease	128 (12)	34 (17)	22 (11)	28 (13)	19 (9)	25 (12)	0.217	
RD	16 (2)	4 (2)	2 (1)	4 (2)	0 (0)	6 (3)	0.108	
PVD	70 (7)	17 (8)	15 (7)	17 (8)	14 (7)	7 (3)	0.271	
CPD	120 (12)	26 (13)	19 (9)	24 (11)	20 (10)	31 (15)	0.37	
Hypertension	275 (27)	60 (29)	64 (31)	58 (28)	52 (25)	41 (20)	0.088	
**Vital signs**								
Mean HR (min^− 1^)	78.2 (70.06, 87.87)	73.27 (66.88, 82.84)	74.78 (68.95, 84.37)	79.06 (70.29, 87.62)	80.96 (72, 89.73)	84 (74.14, 94.42)	< 0.001	
Mean AP (mmHg)	85.83 ± 10.03	85.96 ± 9.95	86.77 ± 9.19	86.64 ± 10.7	86.08 ± 10.29	83.72 ± 9.74	0.015	
Mean RR (min^− 1^)	18.35 (16.79, 20.21)	18.1 (16.5, 19.68)	18.12 (16.54, 20.21)	18.42 (16.94, 19.88)	18.44 (16.31, 20.72)	18.72 (17.29, 20.64)	0.033	
Mean Temp (°C)	36.94 (36.73, 37.24)	36.87 (36.69, 37.1)	36.92 (36.73, 37.22)	36.95 (36.73, 37.24)	37 (36.78, 37.28)	36.99 (36.77, 37.36)	0.001	
Mean SpO2 (%)	97.08 (95.92, 98.34)	96.93 (96.05, 98)	96.9 (95.8, 98.08)	96.9 (95.72, 98.23)	97.42 (96.16, 98.38)	97.72 (95.89, 98.96)	0.004	
**Laboratory test**								
WBC (K/uL)	9.95 (7.93, 12.78)	9.75 (7.9, 12.43)	10.22 (8.11, 13.04)	10.05 (8.07, 12.61)	10.2 (8.18, 12.93)	9.78 (7.51, 12.49)	0.233	
Neutrophils (%)	79.9 (70.35, 85.8)	80 (69.42, 86.14)	80.5 (70.47, 86.21)	78.3 (70.25, 84.3)	81.6 (72.75, 86.34)	78.65 (69.54, 85.05)	0.252	
Lymphocytes (%)	25 (15.8, 38.8)	25.75 (16.38, 39.53)	24.4 (15.2, 38.62)	27.2 (17.8, 39.5)	22.3 (14.05, 37.15)	26.9 (17.8, 39.73)	0.132	
Hemoglobin (g/dL)	12.7 (11.45, 13.8)	12.7 (11.23, 13.78)	12.4 (11.35, 13.57)	12.95 (11.7, 14.16)	12.5 (11.38, 13.8)	12.75 (11.45, 13.8)	0.05	
Platelets (K/uL)	209 (171, 257.88)	197.5 (165.75, 260.25)	219.75 (169.88, 276.75)	210 (170.88, 256.25)	209.25 (176.5, 253.38)	205 (172.75, 246.38)	0.274	
RDW (%)	13.6 (13, 14.49)	13.65 (13.1, 14.35)	13.65 (13.1, 14.7)	13.55 (12.9, 14.31)	13.5 (12.9, 14.5)	13.68 (13, 14.49)	0.299	
Calcium (mg/dL)	8.8 (8.4, 9.2)	8.8 (8.35, 9.2)	8.78 (8.4, 9.15)	8.9 (8.51, 9.3)	8.85 (8.33, 9.24)	8.85 (8.55, 9.15)	0.358	
Sodium (mEq/L)	139.5 (137.5, 142)	140 (137.75, 142)	139.5 (137, 141.5)	140 (137.5, 142)	139.5 (137.5, 142)	139.5 (137.88, 142)	0.858	
Potassium (mEq/L)	4 (3.7, 4.3)	3.95 (3.7, 4.3)	4.05 (3.75, 4.34)	4 (3.7, 4.31)	4 (3.75, 4.3)	3.92 (3.7, 4.3)	0.701	
Creatinine (mg/dL)	0.9 (0.75, 1.1)	0.95 (0.75, 1.1)	0.9 (0.75, 1.15)	0.9 (0.7, 1.1)	0.9 (0.74, 1.1)	0.9 (0.75, 1.1)	0.123	
BUN (mg/dL)	16.5 (13, 21.5)	17 (13, 21.5)	16.5 (13, 21.5)	16 (12, 20.5)	17 (12.5, 22.12)	16.5 (13, 21)	0.679	
Bilirubin (mg/dL)	0.6 (0.4, 0.9)	0.6 (0.4, 0.9)	0.6 (0.4, 0.8)	0.6 (0.5, 0.9)	0.6 (0.4, 0.9)	0.7 (0.5, 0.9)	0.685	
ALT ^c^	1.28 (1.18, 1.45)	1.3 (1.15, 1.48)	1.3 (1.18, 1.45)	1.28 (1.15, 1.45)	1.28 (1.18, 1.45)	1.28 (1.17, 1.4)	0.929	
AST ^d^	1.41 (1.3, 1.58)	1.42 (1.32, 1.6)	1.41 (1.28, 1.57)	1.41 (1.28, 1.58)	1.41 (1.3, 1.59)	1.41 (1.32, 1.56)	0.711	
PT (s)	12.4 (11.5, 13.8)	12.5 (11.6, 13.75)	12.4 (11.62, 14.07)	12.32 (11.5, 14)	12.3 (11.51, 13.65)	12.4 (11.36, 13.69)	0.685	
APTT (s)	27.95 (25.45, 30.85)	28.2 (25.32, 30.9)	28.1 (25.7, 30.65)	27.7 (25.59, 31.97)	27.95 (25, 30.35)	27.92 (25.98, 30.63)	0.537	
TG (mg/dL)	94 (71, 138)	100 (74, 147.25)	99 (72, 140)	93 (67.5, 139.25)	98 (70.75, 133.75)	91 (69.25, 120.38)	0.438	
ABG (mg/dL)	124 (103, 155)	96 (89, 109)	108 (98, 119)	121 (111, 137)	136.5 (125.75, 153.25)	181 (156, 239.5)	< 0.001	
HbA1C (%)	5.7 (5.4, 6.3)	6 (5.6, 6.7)	5.7 (5.38, 6.1)	5.7 (5.4, 6.3)	5.6 (5.3, 6)	5.7 (5.2, 6.68)	< 0.001	
**Events**								
Los ICU (days)	3.32 (1.73, 7.59)	2.07 (1.14, 4.95)	3.22 (1.66, 6.65)	3.31 (1.78, 6.84)	4.24 (1.92, 8.09)	4.76 (2.04, 9.61)	< 0.001	
Los hospital (days)	7.63 (4.05, 14.82)	5.78 (3.65, 12)	7.66 (3.92, 13.15)	7.02 (4.46, 13.64)	8.72 (4.92, 16.27)	9.75 (4.76, 16.8)	0.002	
30-day mortaility (%)	195 (19)	23 (11)	22 (11)	36 (17)	39 (19)	75 (36)	< 0.001	
1-year mortality (%)	323 (31)	52 (25)	48 (24)	65 (31)	58 (28)	100 (49)	< 0.001	
Lung infection (%)	168 (16)	26 (13)	25 (12)	30 (14)	36 (18)	51 (25)	168 (16)	
**Medications**								
Vasoactive drugs	148 (14)	29 (14)	30 (15)	27 (13)	35 (17)	27 (13)	0.748	
RRT	20 (2)	3 (1)	5 (2)	5 (2)	6 (3)	1 (0)	0.334	
Invasive ventilation	384 (37)	70 (34)	80 (39)	70 (33)	88 (43)	76 (37)	0.242	
Vent time (hours)	40.34 (22.5, 108.87)	34.6 (18.87, 139.9)	37.7 (22, 92)	43.51 (27.15, 112.75)	41.9 (25.5, 97.09)	43.23 (22.45, 123.53)	0.921	

^a^ Continuous data is presented as median (interquartile range), whereas categorical data is presented as frequency (percentage)

^b^ SHR: Q1 (≤ 0.234), Q2 (0.234–0.892), Q3 (0.892–1.033), Q4 (1.033–1.228), Q5 (>1.228)

^c^ ALT in the table is the value after logarithmic transformation

^d^ AST in the table is the value after logarithmic transformation

Abbreviation: SHR, stress hyperglycemia ratio; GCS, Glasgow coma scale; APSIII, acute physiology score III; SAPSII, simplifed acute physiological score II; SOFA, sequential organ failure assessment; OASIS, oxford acute severity of illness score; MI, myocardial infarct; CHF, congestive heart failure; RD, rheumatic disease; PVD, peripheral vascular disease; CPD, chronic pulmonary disease; HR: heart rate; AP: arterial pressure; RR: respiratory rate; Temp: body temperature; WBC, white blood cell count; RDW, red cell distribution width; BUN, blood urea nitrogen; ALT, alanine aminotransferase; AST, aspartate aminotransferase; PT, prothrombin time; APTT, activated partial thromboplastin time; TG, triglycerides; ABG, admission blood glucose; HbA1C, glycated hemoglobin A1

### Association between SHR, ABG and the 30-day mortality

**Fig. 2:**
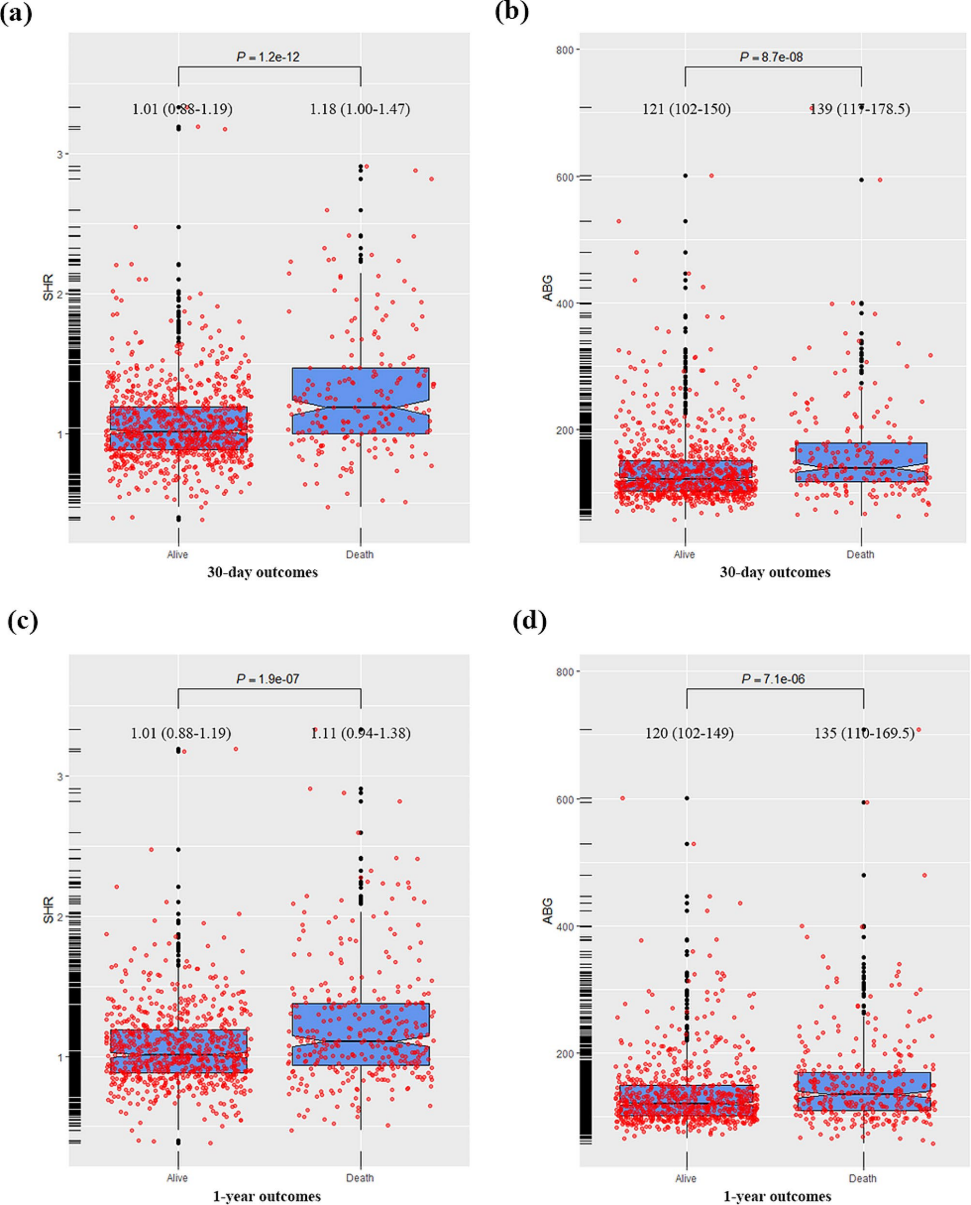
The boxplot of SHR and ABG stratified by primary outcomes. (**a**)~(**c**) The level of SHR and ABG in the 30-day survivors and non-survivors. (**d**)~(**f**) The level of SHR and ABG in the 1-year survivors and non-survivors

**Fig. 3:**
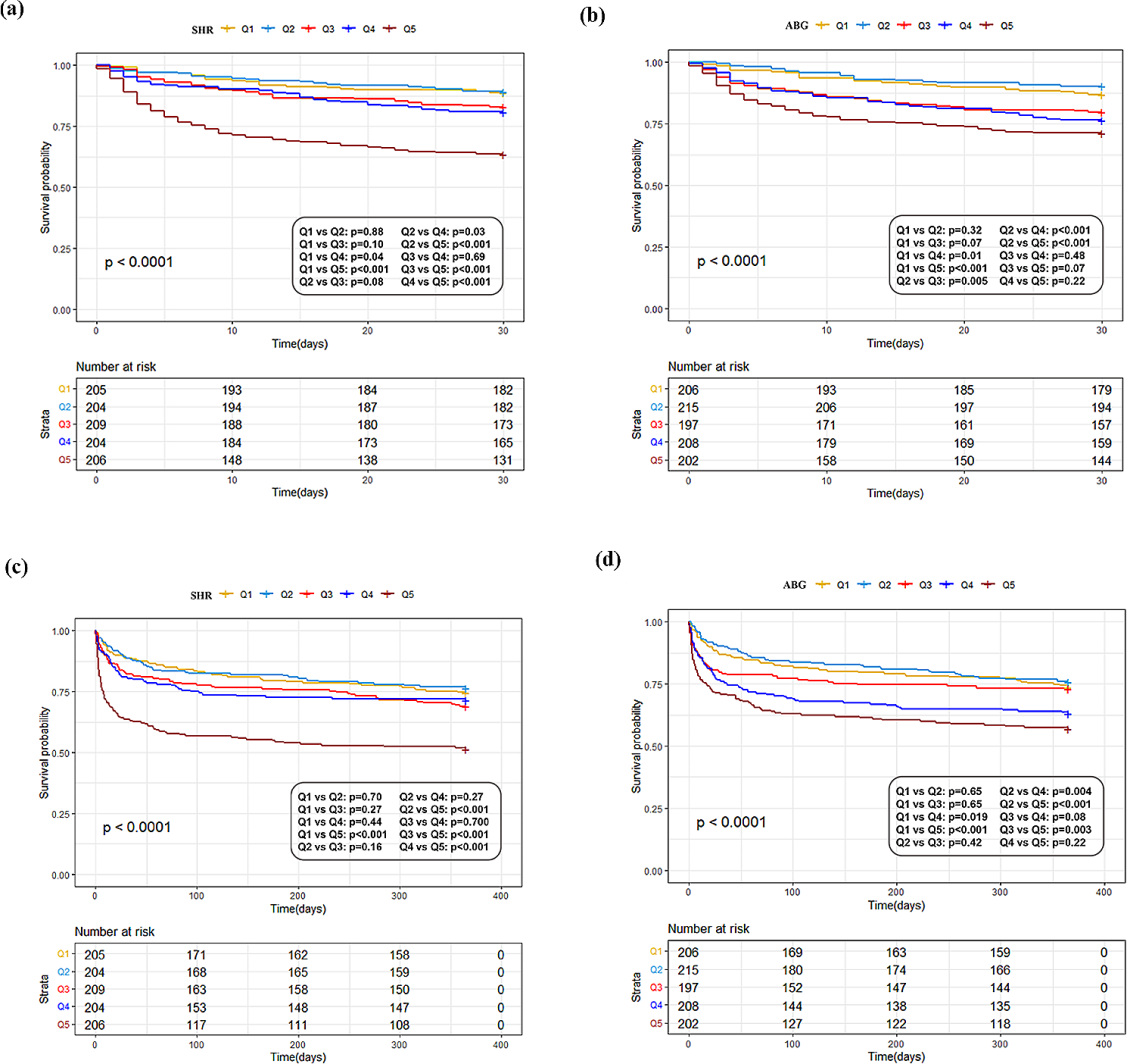
The Kaplan-Meier’s survival curve estimated of critically ill patients with ICH grouped by the quintile of SHR and ABG. (**a**) the 30-day survival analysis of patients divided by the quartiles of SHR (Log-rank *p* < 0.0001). (**b**) the 30-day survival analysis of patients divided by the quartiles of ABG (Log-rank *p* < 0.0001). (**c**) the 1-year survival analysis of patients divided by the quartiles of SHR (Log-rank *p* < 0.0001). (**d**) the 1-year survival analysis of patients divided by the quartiles of ABG (Log-rank *p* < 0.0001)

The levels of SHR and ABG in the 30-day non-survivors was significantly higher than that in the 30-day survivors (1.18 (IQR:1.00-1.47) vs. 1.01 (IQR:0.88–1.19), *p* < 0.001 for SHR; 139 (IQR:117-178.5) vs.121 (IQR:102–150), *p* < 0.001 for ABG) (Fig. 2). The Kaplan-Meier’s survival analysis combined with log-rank test exhibited significant difference in the 30-day mortality among patients divided by the quintile of SHR and ABG (*p* < *0.001* for both). The comparison between groups showed that the 30-day mortality was significantly higher in patients with an SHR in the fifth interval (Q5) than those in the other four intervals (Q1 ~ Q4 vs. Q5: *p* < *0.001)*, but no difference was observed between patients in Q5 and those in Q3 and Q4 of ABG (Q1 ~ Q2 vs. Q5: *p* < 0.001; Q3 vs. Q5: *p* = 0.07; Q4 vs. Q5: *p* = *0.22)* (Fig. 3).

**Fig. 4:**
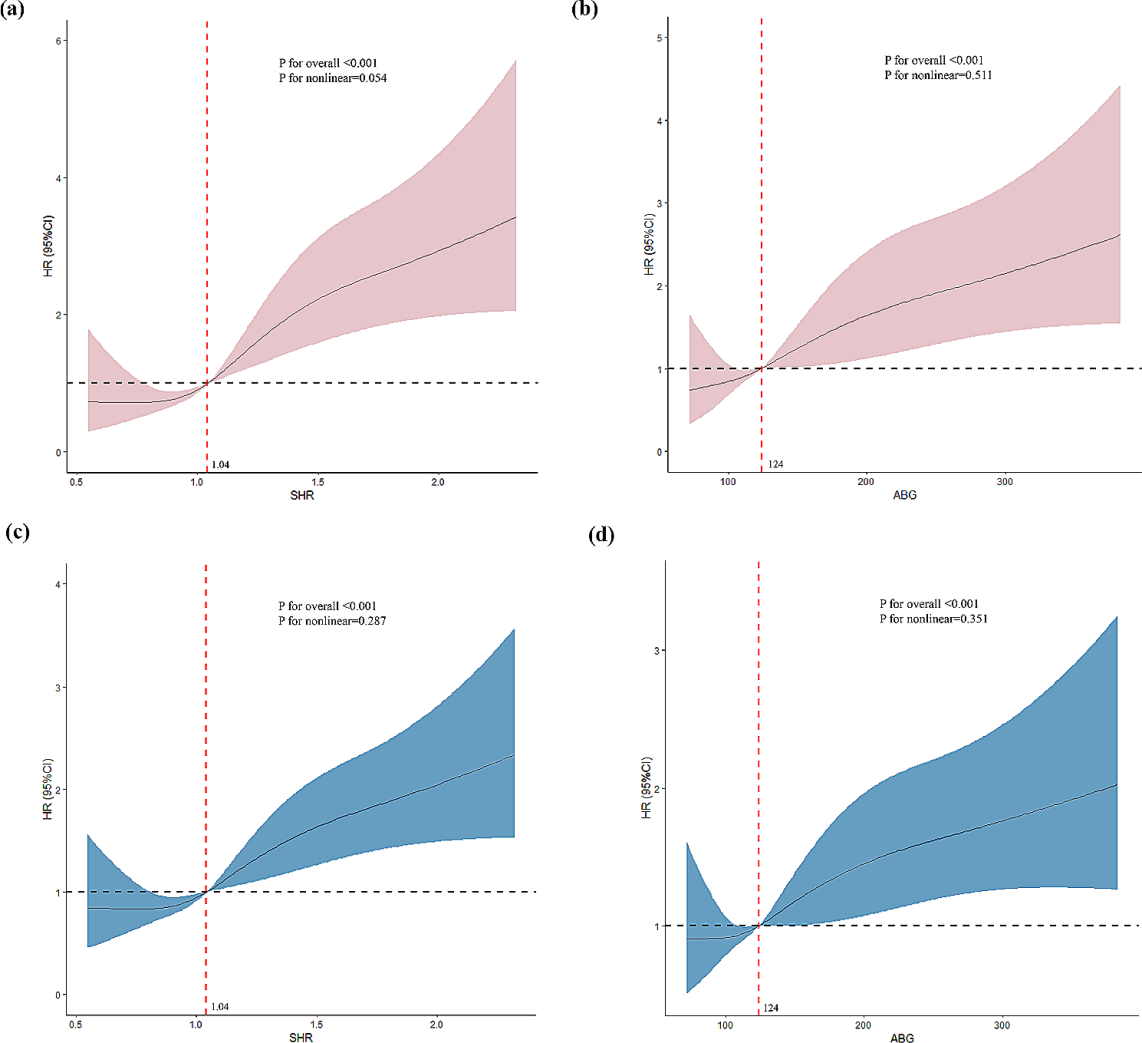
Restricted cubic spline curve for the Hazard ratio of SHR and ABG. SHR 1.04 and ABG 124, represented by the vertical dotted lines, was an estimated hazard ratio of 1.0. The horizontal dotted line represents the hazard ratio of 1.0. (**a**) Restricted cubic spline of SHR for the 30-day mortality. (**b**) Restricted cubic spline of ABG for the 30-day mortality. (**c**) Restricted cubic spline of SHR for the 1-year mortality. (**d**) Restricted cubic spline of ABG for the 1-year mortality

In the adjusted model of Cox regression analysis, patients in Q4 and Q5 of SHR had significantly higher risk of the 30-day death compared with those in Q1 (Q4 vs. Q1: adjusted HR 1.88, 95%CI 1.08–3.27; Q5 vs. Q1: adjusted HR 3.33, 95%CI 2.01–5.51), but no significant difference was observed between patients in Q4 and Q1 of ABG (Q4 vs. Q1: adjusted HR 1.58, 95% CI 0.96–2.59; Q5 vs. Q1: adjusted HR 2.21, 95%CI 1.37–3.57) (Table 2). Furthermore, restricted cubic spline analysis after fully adjusted demonstrated a nearly linear association of SHR and ABG with the risk of 30-day mortality (p for non-linear = 0.054, p for overall < 0.001 for SHR; p for non-linear = 0.511, p for overall < 0.001 for ABG) (Fig. 4).

**Table 2:** Hazard ratio (HR) for all-cause 30-day or 1-year mortality of SHR, ABG and HbA1C in the whole cohort

Variables	30-day mortality								1-year mortality						
	Unadjusted model				Adjusted model ^d^				Unadjusted model				Adjusted model ^e^		
	HR(95% CI)	*p* value	*p*-trend		HR(95% CI)	*p* value	*p*-trend		HR(95% CI)	*p* value	*p*-trend		HR(95% CI)	*p* value	*p*-trend
**SHR**															
Continuous variable per unit	3.41 (2.64,4.40)	< 0.001			2.78 (2.00,3.86)	< 0.001			2.65 (2.10,3.33)	< 0.001			2.00 (1.52,2.64)	< 0.001	
Quantile ^a^			< 0.001				< 0.001				< 0.001				< 0.001
Q1 (*n* = 205)	Ref								Ref						
Q2 (*n* = 204)	0.96 (0.53,1.72)	0.884			1.03 (0.57,1.88)	0.920			0.93 (0.63,1.37)	0.698			0.96 (0.64,1.44)	0.851	
Q3 (*n* = 209)	1.59 (0.94,2.68)	0.084			1.63 (0.94,2.82)	0.080			1.28 (0.89,1.85)	0.179			1.26 (0.86,1.84)	0.233	
Q4 (*n* = 204)	1.78 (1.06,2.98)	0.028			1.88 (1.08,3.27)	0.027			1.19 (0.82,1.73)	0.356			1.23 (0.82,1.83)	0.315	
Q5 (*n* = 206)	3.95 (2.48,6.31)	< 0.001			3.33 (2.01,5.51)	< 0.001			2.49 (1.78,3.48)	< 0.001			2.09 (1.46,3.00)	< 0.001	
**ABG**															
Continuous variable per unit	1.01 (1.00,1.02)	< 0.001			1.00 (1.00,1.01)	< 0.001			1.01 (1.00,1.02)	< 0.001			1.01 (1.00,1.01)	< 0.001	
Quantile ^b^			< 0.001				< 0.001				< 0.001				< 0.001
Q1 (*n* = 206)	Ref								Ref						
Q2 (*n* = 215)	0.73 (0.42,1.30)	0.289			0.81 (0.45,1.46)	0.484			0.91 (0.62,1.33)	0.613			1.06 (0.71,1.56)	0.789	
Q3 (*n* = 197)	1.65 (1.02,2.70)	0.043			1.35 (0.89,2.31)	0.119			1.09 (0.75,1.60)	0.641			1.27 (0.85,1.89)	0.243	
Q4 (*n* = 208)	1.92 (1.20,3.07)	0.006			1.58 (0.96,2.59)	0.073			1.57 (1.11,2.22)	0.011			1.29 (0.90,1.86)	0.171	
Q5 (*n* = 202)	2.51 (1.59,3.97)	< 0.001			2.21 (1.37,3.57)	0.001			1.97 (1.40,2.77)	< 0.001			1.79 (1.26,2.54)	0.001	

^a^ SHR: Q1 (≤ 0.863), Q2 (0.863–0.978), Q3 (0.978–1.112), Q4 (1.112–1.314), Q5 (>1.314)

^b^ Glucose: Q1 (≤ 99.4), Q2 (99.4–116), Q3 (116–134), Q4 (134–167), Q5 (>167)

^c^ HbA1C: Q1 (≤ 5.3), Q2 (5.3–5.6), Q3 (5.6–5.9), Q4 (5.9–6.6), Q5 (>6.6)

^d^ Adjusted model was adjusted for the variables with a *p* value < 0.01 in the univariable Cox regression, including gender, age, heart rate, mean arterial pressure, respiratory rate, body temperature, SpO_2_, Glasgow Coma Scale, white blood cell count, neutrophil percentage, lymphocyte percentage, hemoglobin, red blood cell distribution width, serum calcium, serum sodium, creatinine, blood urea nitrogen, aspartate aminotransferase, and bilirubin

^e^ Adjusted model was adjusted for the variables with a *p* value < 0.01 in the univariable Cox regression, including gender, age, myocardial infarct, congestive heart failure, renal disease, heart rate, mean arterial pressure, respiratory rate, body temperature, SpO_2_, Glasgow Coma Scale, white blood cell count, lymphocyte percentage, hemoglobin, red blood cell distribution width, serum calcium, serum sodium, creatinine, blood urea nitrogen, aspartate aminotransferase, bilirubin, and prothrombin time

### Association between SHR, ABG and the 1-year mortality

The levels of SHR and ABG in the 1-year non-survivors was significantly higher than that in the 1-year survivors (1.11 (IQR:0.94–1.38) vs. 1.01 (IQR:0.88–1.19), *p* < 0.001 for SHR; 135 (IQR:110-169.5) vs. 120 (IQR:102–149), *p* < 0.001 for ABG) (Fig. 2). The Kaplan-Meier’s survival analysis combined with log-rank test showed significant difference in the 1-year survival among patients divided by the quintile of SHR and ABG (*p* < *0.001* for both). The comparison between groups showed that patients’ 1-year mortality was significantly higher in Q5 of SHR than that in the other four intervals (Q1 ~ Q4 vs. Q5: *p* < *0.001)*, but no significant difference was observed between patients in Q5 and Q4 of ABG (Q1 ~ Q2 vs. Q5: *p* < 0.001, Q3 vs. Q5: *p* = 0.003, Q4 vs. Q5: *p* = 0.22 for ABG*)* (Fig. 3).

In the adjusted model of Cox regression analysis, patients in Q5 of SHR and ABG had significantly higher risk of the 1-year mortality compared with those in Q1 (adjusted HR 2.09, 95% CI 1.46-3.00 for SHR; adjusted HR 1.79, 95% CI 1.26–2.54 for ABG) (Table 2). Moreover, restricted cubic spline analysis after fully adjusted demonstrated a nearly linear association of SHR and ABG with the risk of 1-year mortality (p for non-linear = 0.287, p for overall < 0.001 for SHR; p for non-linear = 0.351, p for overall = 0.001 for ABG) (Fig. 4).

### Association of SHR and ABG with the primary outcomes in the diabetic and non-diabetic subgroups

To further assess the impact of glucose metabolism status on the risk stratification value of SHR and ABG for the 30-day and 1-year outcomes of ICH, Cox regression analysis was conducted in the diabetic and non-diabetic subgroups.

In the non-diabetic subgroup, patients in Q5 of SHR and ABG had significantly higher risk of 30-day and 1-year mortality compared with those in Q1 (30-day mortality: adjusted HR 4.55, 95%CI 2.33–8.88 for SHR; adjusted HR 2.71, 95%CI 1.55–4.73 for ABG. 1-year mortality: adjusted HR 3.06, 95%CI 1.93–4.86 for SHR; adjusted HR 2.38, 95%CI 1.56–3.63 for ABG). Moreover, significantly higher risk of 30-day and 1-year mortality was also existed in Q4 of SHR compared with Q1 (30-day mortality: adjusted HR 2.92, 95%CI 1.46–5.84. 1-year mortality: adjusted HR 2.02, 95%CI 1.24–3.28). (Additional file 5: Table S3).

In the diabetic subgroup, the risk of 30-day and 1-year mortality had no significant difference between patients in Q2 ~ Q5 of SHR and ABG and those in Q1. However, as continuous variable, SHR was significantly associated with the 30-day and 1-year mortality in the adjusted model, but that was not the case for ABG (30-day mortality: adjusted HR 2.63, 95%CI 1.50–4.60 for SHR; adjusted HR 1.00, 95%CI 0.99–1.01 for ABG. 1-year mortality: adjusted HR 2.12, 95%CI 1.33–3.39 for SHR; adjusted HR 1.00, 95%CI 0.99–1.01 for ABG) (Table 3).

**Table 3:** Hazard ratio (HR) for all-cause 30-day or 1-year mortality of SHR and ABG in the patients with diabetes

Variables	30-day mortality								1-year mortality						
	Unadjusted model				Adjusted model ^c^				Unadjusted model				Adjusted model ^d^		
	HR(95% CI)	*p* value	*p*-trend		HR(95% CI)	*p* value	*p*-trend		HR(95% CI)	*p* value	*p*-trend		HR(95% CI)	*p* value	*p*-trend
**SHR**															
Continuous variable per unit	2.95 (1.82,4.77)	< 0.001			2.63 (1.50,4.60)	< 0.001			2.04 (1.34,3.10)	< 0.001			2.12 (1.33,3.39)	0.002	
Quantile ^a^			0.003				0.042				0.050				0.132
Q1 (*n* = 60)	Ref								Ref						
Q2 (*n* = 62)	0.57 (0.21,1.56)	0.270			0.65 (0.23,1.87)	0.430			0.66 (0.33,1.31)	0.235			0.67 (0.33,1.39)	0.288	
Q3 (*n* = 60)	0.79 (0.31,2.00)	0.621			0.58 (0.22,1.53)	0.268			0.94 (0.50,1.75)	0.837			0.69 (0.35,1.34)	0.273	
Q4 (*n* = 61)	0.99 (0.41,2.37)	0.974			1.01 (0.41,2.52)	0.975			0.90 (0.48,1.70)	0.745			1.14 (0.58,2.25)	0.701	
Q5 (*n* = 61)	2.27 (1.06,4.86)	0.034			1.59 (0.72,3.52)	0.254			1.53 (0.86,2.75)	0.150			1.26 (0.67,2.37)	0.469	
**ABG**															
Continuous variable per unit	1.00 (1.00,1.01)	0.007			1.00 (0.99,1.01)	0.350			1.00 (0.99,1.01)	0.103			1.01 (0.99,1.01)	0.232	
Quantile ^b^			0.008				0.010				0.074				0.254
Q1 (*n* = 61)	Ref								Ref						
Q2 (*n* = 62)	1.40 (0.56,3.47)	0.472			1.06 (0.41,2.74)	0.912			0.90 (0.47,1.73)	0.756			0.61 (0.30,1.23)	0.167	
Q3 (*n* = 60)	1.03 (0.39,2.73)	0.959			0.86 (0.31,2.39)	0.779			1.08 (0.58,2.03)	0.802			1.22 (0.62,2.39)	0.563	
Q4 (*n* = 60)	1.05 (0.39,2.79)	0.927			0.98 (0.36,2.67)	0.963			0.81 (0.41,1.60)	0.543			0.85 (0.42,1.72)	0.657	
Q5 (*n* = 61)	2.72 (1.19,6.21)	0.018			2.38 (0.99,5.76)	0.054			1.63 (0.90,2.95)	0.104			2.01 (0.96,3.83)	0.234	

^a^ SHR: Q1 (≤ 0.833), Q2 (0.833–0.981), Q3 (0.981–1.162), Q4 (1.162–1.409), Q5 (>1.409)

^b^ ABG: Q1 (≤ 119.6), Q2 (119.6–146), Q3 (146–179), Q4 (179-238.8), Q5 (>238.8)

^c^ Adjusted model was adjusted for the variables with a *p* value < 0.01 in the univariable Cox regression, including gender, age, mean arterial pressure, SpO_2_, neutrophil percentage, lymphocyte percentage, hemoglobin, and red blood cell distribution width

^d^ Adjusted model was adjusted for the variables with a *p* value < 0.01 in the univariable Cox regression, including gender, age, myocardial infarct, congestive heart failure, renal disease, mean arterial pressure, respiratory rate, SpO_2_, Glasgow Coma Scale, hemoglobin, red blood cell distribution width, serum sodium, creatinine, and blood urea nitrogen

### Predictive value of SHR and ABG for the primary outcomes

Compared with the original Cox regression models of the severity of illness scores (APSIII, SAPSII, SOFA, or OASIS), adding SHR or ABG into the original models can significantly increase the C-statistic for predicting both of the 30-day and 1-year outcomes in the whole cohort and in patients without diabetes (Table 4; Additional file 6: Table S4). In patients with diabetes, adding SHR into the original models can significantly increase the C-statistic for predicting the 30-day outcome, while in terms of the 1-year prognosis, SHR can improve the predictive efficiency of the original model constructed by SOFA or OASIS. Notably, no significant improvement of predictive efficiency for both of the 30-day and 1-year outcomes was observed when adding ABG into anyone of the original models (Additional file 7: Table S5). More importantly, compared with ABG, SHR exhibited higher predictive efficiency and can better improve the C-statistics of the original models regarding 30-day and 1-year outcomes, especially in patients with diabetes (Additional file 8: Table S6; Additional file 9: Table S7).

**Table 4:** Discrimination ability of SHR and ABG for all cause 30-day and 1-year mortality in the whole cohort

	30-day mortality			1-year mortality	
	C-Statistic	*p* value		C-Statistic	*p* value
APSIII	0.684	Ref		0.666	Ref
APSIII + SHR	0.720	< 0.001		0.685	< 0.001
APSIII + ABG	0.699	0.003		0.674	0.023
SAPSII	0.729	Ref		0.724	Ref
SAPSII + SHR	0.761	< 0.001		0.739	< 0.001
SAPSII + ABG	0.742	< 0.001		0.729	0.002
SOFA	0.672	Ref		0.660	Ref
SOFA + SHR	0.723	< 0.001		0.685	< 0.001
SOFA + ABG	0.697	< 0.001		0.671	0.001
OASIS	0.703	Ref		0.680	Ref
OASIS + SHR	0.738	< 0.001		0.697	< 0.001
OASIS + ABG	0.714	< 0.001		0.683	< 0.001

SHR, stress hyperglycemia ratio; ABG, admission blood glucose; APSIII, acute physiology score III; SAPSII, simplified acute physiological score II; SOFA, sequential organ failure assessment; OASIS, oxford acute severity of illness score

## Discussion

### Main findings

This study, for the first time, compared the prognostic effect and predictive value of ABG and SHR for the short- and long-term outcomes in critically ill patients with ICH, and two main findings have been obtained. First, SHR, instead of ABG, was an independent risk factor of the 30-day and 1-year mortality of ICH in the diabetic population. Second, SHR might exhibited higher predictive efficiency for the 30-day and 1-year mortality of ICH than ABG, especially in the diabetic cohort.

### Findings of previous studies and contribution of this study

Since an appropriate predictor should be effectively intervened through existing management to improve the outcome, it is important to determine whether elevated ABG after ICH is associated with increased poor outcomes. Evidence from two large-scale, multi-center studies supports ABG as an independent predictor of poor outcome in patients with ICH [5, 18], but the prognostic effects of ABG based on patients’ diabetic status is not elucidated in these studies due to the lack of direct comparison between diabetics and non-diabetics. Based on this deficiency, Sun et al. conducted a multi-center, prospective cohort study which indicated that elevated ABG is an independent predictor of the 3-month poor outcome in ICH patients and its prognostic value is greater in non-diabetics than diabetics [14]. Besides, an earlier study also found that high ABG in nondiabetics was a significant predictor of death during the first 28 days of the onset of ICH [19]. Consistent with these findings, our study proved that ABG was positively associated with higher 30-day and 1-year mortality in the whole study cohort with ICH and in the non-diabetic subgroup.

A predominant defect that may restrict the clinical practice of ABG is that the level of ABG may be influenced by a number of factors, such as diabetic status, recent glycemia control status, etc. Therefore, the rise of blood glucose due to ICH compared with the background status may be a better predictor of the outcomes of ICH. However, the level of blood glucose prior to ICH is nearly impossible to obtain, especially in non-diabetic patients. Fortunately, this thorny problem is expected to be overcome by using SHR, an index that more accurately reflects the extent of stress-induced hyperglycemia by correction for chronic glycemic status [20]. A pile of studies had demonstrated that SHR is a useful indicator for predicting the outcomes of acute myocardial infarction [7, 10, 21–23]. Moreover, in a landmark study on the association between SHR and the risk of adverse outcomes in acute ischemic stroke (AIS), Huang et al. incorporated a total of thirteen studies encompassing 184,179 individuals and found that higher SHR was significantly associated with increased risk of various adverse outcomes, including a 2.64-fold increased risk of 3-month poor functional outcomes, a 3.11-fold increased risk of 3 month mortality, and a 2.80-fold increased risk of 1-year mortality [24]. However, evidence about the role of SHR in the outcomes of ICH is still limited. Chu and his colleagues seemed to be the first to investigate the effect of SHR on ICH. They revealed that SHR is independently associated with hematoma expansion and 3-month mortality in ICH patients [11]. Consistent with their findings, our study proved that SHR was positively associated with the risk of short- and long-term mortality in the critically ill patients with ICH. More importantly, we proved that SHR, as a continuous variable, was independently related to the 30-day and 1-year mortality in ICH patients with diabetes, but that was not the case for ABG. This result was similar to a recent study derived from the Chinese Cerebral Hemorrhage: Mechanisms and Intervention Study (CHEERY) [25]. In this study, Chen et al. divided ICH patients into nondiabetic normoglycemia (NDN), diabetic normoglycemia (DN), diabetic hyperglycemia (DH), and stress-induced hyperglycemia (SIH) groups, and found that compared with patients with NDN, DH did not increase the risk of poor outcome and mortality, whereas SIH was an independent risk factor for pulmonary infection and 30- and 90-day death after ICH [25]. Although Chen et al. defined stress hyperglycemia by other method (having a diabetes history or HbA1c ≥ 6.5%, and admission blood glucose ≥ 7.8 mmol/L) [26] instead of SHR, the results from their study and ours indicated that stress hyperglycemia may be more closely associated with adverse prognosis in diabetic patients with ICH than admission hyperglycemia. In addition, we revealed that SHR had higher predictive value than ABG respect to the 30-day and 1-year outcomes, especially in patients with diabetes, further supporting SHR as a more reliable and useful indicator than ABG in the risk stratification and prognosis prediction of diabetic patients with ICH.

### Detrimental effects of stress hyperglycemia and its mechanisms

The effects of stress hyperglycemia on ICH are complex and not fully understood. Stress hyperglycemia has been shown to promote the generation of oxygen-free radical, which can then induce toxic effect and lead to neuronal apoptosis in experimental ICH modle [27, 28]. It is reported that hyperglycemia after ICH is associated with neutrophil and lymphocyte ratio, indicating that stress hyperglycemia may induce inflammatory responses and then promote secondary brain injury. Moreover, studies have also shown that the superoxide production induced by hyperglycemic can disrupt the blood–brain barrier (BBB) and aggravate cerebral edema in rat models [29]. Hematoma expansion has been considered as an important contributor to the worsening prognosis of ICH, studies has revealed that stress hyperglycemia leads to hematoma expansion possibly by impairing vessels integrity nearby the site of initial bleeding and increasing the expression of nuclear factor kappa B and matrix metalloproteinase-9 [30]. Meanwhile, Aquaporin-4 (AQP4) is the most abundant aquaporin in the brain that protects neuron from apoptosis by alleviating BBB disruption and brain edema [31, 32]. Studies had demonstrated that hyperglycemia may exacerbate outcome of ICH through downregulating expression of AQP4 [33]. In addition, by using magnetic resonance imaging with spectroscopy, scholars had found an association of hyperglycemia and brain lactate with penumbral damage in patients with ischemic stroke, therefore it is reasonable to infer that there may be a similar injury mechanism in ICH [34].

### Mechanisms of the different prognostic values of ABG

Our data exhibited different prognostic values of elevated ABG in ICH patients with and without diabetes, which was consistent with previous findings on ACS or AMI [8, 35]. It is difficult to clearly explain this between-group difference in the effect of ABG, but several possible mechanisms may contribute to it. First, undiagnosed diabetes or pre-diabetes may be existed in some non-diabetic patients with elevated ABG. This part of patients may represent a higher-risk cohort with poor outcomes, especially in those with glucose ≥ 11.1 mmol/L. Second, as studies had shown that insulin resistance is a marker of increased risk of incident ischemic stroke in the nondiabetic individuals [36, 37], it is reasonable to infer that insulin resistance may exist in non-diabetic patients with ICH, particular in those with elevated ABG. Compared with the diabetic patients with hyperglycemia during ICH, the non-diabetic ones may expose to a greater degree of fluctuation in insulin resistance in order to reach the same level of hyperglycemia, which may result in higher risk of adverse outcome. Third, the non-diabetics may suffer from greater degree of stress reactions to reach the same hyperglycemic state as their diabetic counterparts, which may lead to higher mortality in ICH patients without diabetes [38]. It is worth noting that we focused on critically ill patients in this study. In a state of severe illness, patients’ body can enter a maladaptive state of allostatic overload, which may amplify the adverse effects of hyperglycemia mentioned above [39]. Forth, elevated ABG in the non-diabetic patients was always overlooked during hospitalization. A better management of elevated ABG in the diabetic patients compared with their non-diabetic counterparts may result in different outcomes.

### Implications for glucose management in ICH

Glucose monitoring and management is often considered as a part of the general care of all patients, including those with ICH. In the NICE-SUGAR trial, a blood glucose target of < 180 mg/dL was associated with lower mortality than a target of 81 to 108 mg/dL, suggesting that targets for treating hyperglycemia should be less intensive in critically ill patients [40]. Mauro and colleagues reported that tight systemic glycemic control (80–110 mg/dL) may impair cerebral glucose metabolism after severe brain injury, which in turn correlates with increased mortality [41]. These evidences support a viewpoint that intensive insulin therapy, which has been proved to be beneficial in patients with ischemic myocardium, may increase the risk of hypoglycemic events and worsen outcomes in ICH patients. However, the optimal glucose level at which treatment should be initiated and the target range of treatment are still unclear. One reason for this confusion, which has been stated by the American Stroke Association, is that the relationship among serum glucose, the timing of that measurement, and the presence/absence of comorbid diabetes remains unclear [42–45]. Most of the previous studies take advantage of ABG or systemic glucose as the target of their intervention, but as the level of ABG or systemic glucose can be influenced by a number of factors, such as different diabetic status or insulin resistance levels, these two indicators may not be the good choice to reflect the acute glycemic status. In this case, SHR may be better to identify the true stress hyperglycemia by correction for chronic glycemic status. Evidence from this study also supports SHR as a better prognostic indicator for patients with ICH, especially for those with diabetes. Therefore, a more personalized strategy that based on patients’ diabetic status and combined SHR and ABG as the target may be a new choice for glucose management in patients with ICH. But this idea needs to be further confirmed in the future by high-quality randomized controlled studies.

### Limitations of the study

Several limitations of this study should be concerned. First, the retrospective nature of the observational study determined that although multivariable-adjusted analysis was conducted, unmeasured variables could impact outcomes. Second, we excluded ICH patients with missing data of ABG and HbA1c, but if the pattern of missing is not completely random, selection bias may compromise the reliability of the conclusions. Third, as the value of admission glucose is also subjected to meal timing, fasting plasma glucose may be superior to reflect the true state of blood glucose, but this indicator cannot be collected from the MIMIC-IV database. Fourth, both SHR and ABG are derived from the first result of blood glucose test since ICU admission, which cannot fully reflect the profile of glucose fluctuation in the acute phase of ICH. Therefore, the association between glucose or SHR variation identified by continuous glucose monitoring and prognosis of ICH need to be evaluated in the future. Last but not least, as a single-center study, the conclusions of this study cannot be directly extrapolated to general population without validating in more centers.

## Conclusion

This study demonstrated that SHR might be a more useful and reliable marker than ABG for prognostic prediction and risk stratification in critically ill patients with ICH, especially in those without diabetes. Further prospective studies with larger population are needed to confirm the findings and determine whether glucose management targeting SHR will improve clinical outcomes of critically ill patients with ICH.

### Electronic supplementary material

Below is the link to the electronic supplementary material.

Supplementary Material 1

Supplementary Material 2

Supplementary Material 3

Supplementary Material 4

Supplementary Material 5

Supplementary Material 6

Supplementary Material 7

Supplementary Material 8

Supplementary Material 9

## Data Availability

The datasets used and analyzed during the current study are available from the corresponding author on reasonable request.

## Abbreviations

ABG: Admission blood glucose
ACS: Acute coronary syndrome
ALT: Alanine aminotransferase
AMI: Acute myocardial infarction
APSIII: Acute physiology score III
APTT: Activated partial thromboplastin time
BUN: Blood urea nitrogen
GCS: Glasgow coma scale
HbA1c: Hemoglobin A1c
ICD: International Classification of Diseases
ICH: Intracerebral hemorrhage
MI: Myocardial infarct
MIMIC-IV: The Medical Information Mart for Intensive Care
OASIS: Oxford acute severity of illness score
PT: Prothrombin time
RDW: Red cell distribution width
RRT: Renal replacement treatment
SAPSII: Simplified acute physiological score II
SHR: Stress hyperglycemia ratio
SOFA: Sequential organ failure assessment
VIF: Variance inflation factor
WBC: White blood cell count
