# Supplementary material for: Prognostic significance of the stress hyperglycemia ratio and admission blood glucose in diabetic and nondiabetic patients with spontaneous intracerebral hemorrhage
Source: Diabetol Metab Syndr. 2024 Mar 4;16:58. doi: 10.1186/s13098-024-01293-0 (PMC10910766; doi:10.1186/s13098-024-01293-0)
Supplement: Supplementary file 1 — Supplementary Material 1 [file 13098_2024_1293_MOESM1_ESM.docx]

**Table S1. The International Classification of Diseases, 9th and 10th Revision of the comorbidities included in the study.**

| **Comorbidity** | **ICD-9 code** |  | **ICD-10 code** |
| --- | --- | --- | --- |
| MI | '410', '412', |  | 'I21', 'I22', 'I252' |
|  |  |  |  |
| CHF | '428', '39891', '40201', '40211', '40291', '40401', '40403', '40411', '40413', '40491', '4254', '4255', '4256', '4257', '4258', '4259', |  | 'I43', 'I50', 'I099', 'I110', 'I130', 'I132', 'I255', 'I420', 'I425', 'I426', 'I427', 'I428', 'I429', 'P290' |
|  |  |  |  |
| Diabetes | '2500', '2501', '2502', '2503', '2508', '2509', '2504', '2505', '2506', '2507' |  | 'E100', 'E10l', 'E106', 'E108', 'E109', 'E110', 'E111', 'E116', 'E118', 'E119', 'E120', 'E121', 'E126', 'E128', 'E129', 'E130', 'E131', 'E136', 'E138', 'E139', 'E140', 'E141', 'E146', 'E148', 'E149', 'E102', 'E103', 'E104', 'E105', 'E107', 'E112', 'E113', 'E114', 'E115', 'E117', 'E122', 'E123', 'E124', 'E125', 'E127', 'E132', 'E133', 'E134', 'E135', 'E137', 'E142', 'E143', 'E144', 'E145', 'E147' |
|  |  |  |  |
| Renal disease | '582', '585', '586', 'V56', '5880', 'V420', 'V451', '5830', '5831', '5832', '5833', '5834', '5835', '5836', '5837', '40301', '40311' , '40391', '40402', '40403', '40412', '40413', '40492', '40493' |  | 'N18', 'N19', 'I120', 'I131', 'N032', 'N033', 'N034', 'N035', 'N036', 'N037', 'N052', 'N053', 'N054', 'N055', 'N056', 'N057', 'N250', 'Z490', 'Z491', 'Z492', 'Z940', 'Z992' |
|  |  |  |  |
| RD | '725', '4465', '7100', '7101', '7102', '7103', '7104', '7140', '7141', '7142', '7148' |  | 'M05', 'M06', 'M32', 'M33', 'M34', 'M315', 'M351', 'M353', 'M360' |
|  |  |  |  |
| PVD | '440', '441', '0930', '4373', '4471', '5571', '5579', 'V434', '4431', '4432', '4433', '4434', '4435', '4436', '4437', '4438', '4439' |  | 'I70', 'I71', 'I731', 'I738', 'I739', 'I771', 'I790', 'I792', 'K551', 'K558', 'K559', 'Z958', 'Z959' |
|  |  |  |  |
| CPD | '490', '491', '492', '493', '494', '495', '496', '497', '498', '499', '500', '501', '502', '503', '504', '505', 4168', '4169', '5064', '5081', '5088' |  | 'J40', 'J41', 'J42', 'J43', 'J44', 'J45', 'J46', 'J47', 'J60', 'J61', 'J62', 'J63', 'J64', 'J65', 'J66', 'J67' , 'I278', 'I279', 'J684', 'J701', 'J703' |
|  |  |  |  |
| Hypertension | '4010', '4011', '4019', '40501', '40509', '40511', '40519', '40591', '40599' |  | 'I10 ', 'I15', 'I150', 'I151', 'I152', 'i158', 'I159' |

*Abbreviation:* *MI, myocardial infarct; CHF,* *congestive heart failure; RD,* *rheumatic disease; PVD, peripheral vascular disease; CPD, chronic pulmonary disease.*
