# Supplementary figures and images for: Prognostic significance of the stress hyperglycemia ratio and admission blood glucose in diabetic and nondiabetic patients with spontaneous intracerebral hemorrhage
Source: Diabetol Metab Syndr. 2024 Mar 4;16:58. doi: 10.1186/s13098-024-01293-0 (PMC10910766; doi:10.1186/s13098-024-01293-0)

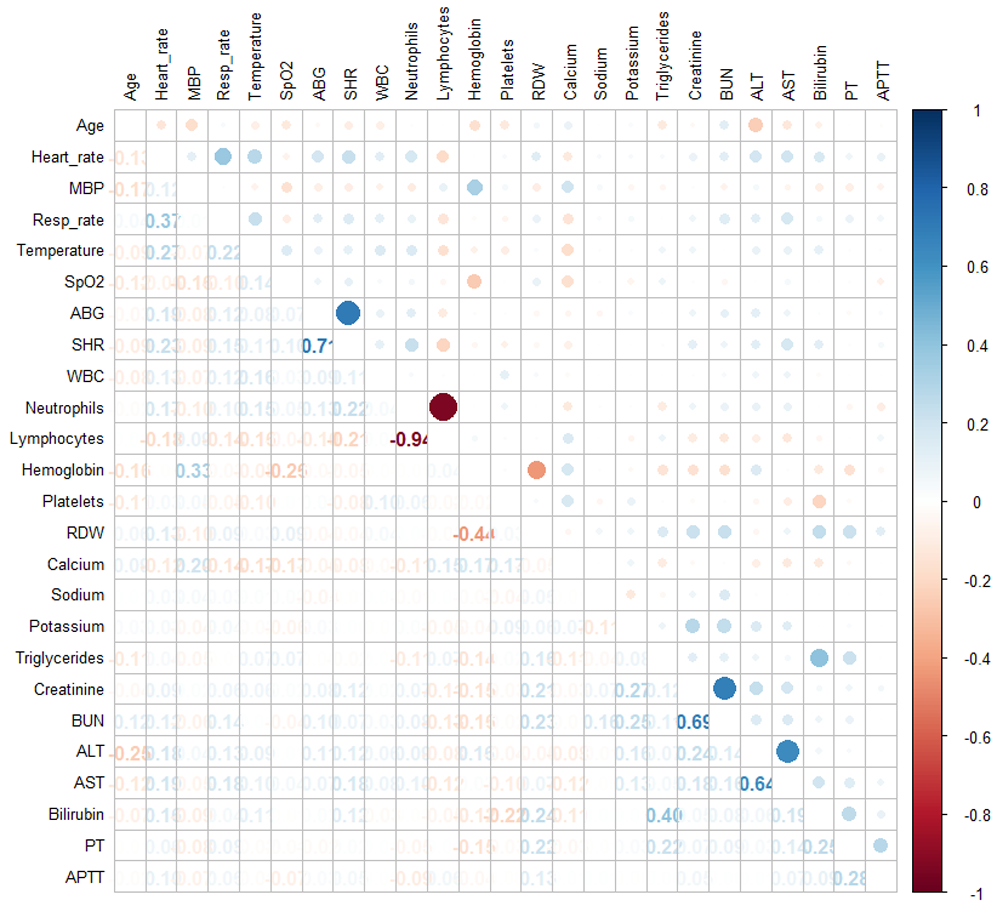

Supplement: Supplementary file 2 — Supplementary Material 2 [file 13098_2024_1293_MOESM2_ESM.tiff]

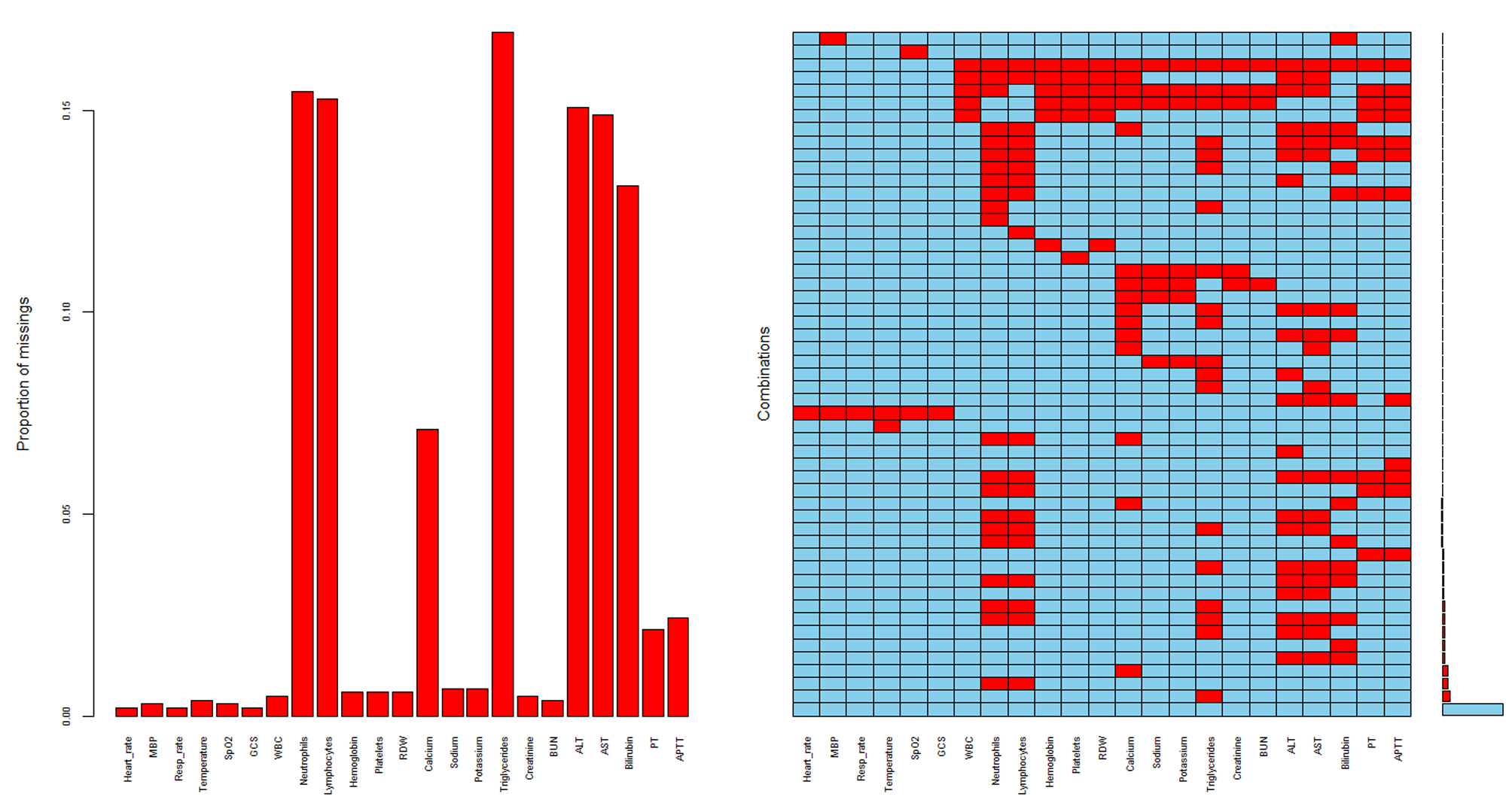

Supplement: Supplementary file 3 — Supplementary Material 3 [file 13098_2024_1293_MOESM3_ESM.tif]
