# Supplementary material for: Prognostic significance of the stress hyperglycemia ratio and admission blood glucose in diabetic and nondiabetic patients with spontaneous intracerebral hemorrhage
Source: Diabetol Metab Syndr. 2024 Mar 4;16:58. doi: 10.1186/s13098-024-01293-0 (PMC10910766; doi:10.1186/s13098-024-01293-0)
Supplement: Supplementary file 4 — Supplementary Material 4 [file 13098_2024_1293_MOESM4_ESM.docx]

**Table S2. Baseline characteristics of participants in diabetic and non-diabetic groups ^a^**

| **Variables** | **Total  (n = 1028)** | **Non-diabetes (n = 695)** | **Diabetes (n = 333)** | ***p-value*** |
| --- | --- | --- | --- | --- |
| Age, years | 71.09 (60.05, 81.97) | 70.57 (58.29, 81.66) | 72.25 (62.53, 82.28) | 0.062 |
| Male, n% | 548 (53) | 367 (53) | 181 (54) | 0.690 |
| GCS | 14 (12, 15) | 14 (12, 15) | 14 (11, 15) | 0.389 |
| **Severe Score** |  |  |  |  |
| APSIII | 34 (25, 44) | 33 (25, 43) | 36 (27, 48) | 0.001 |
| SAPSII | 31 (25, 39) | 31 (24, 38) | 32 (26, 39) | 0.059 |
| SOFA | 3 (1, 4) | 3 (1, 4) | 3 (2, 4) | 0.170 |
| OASIS | 30 (26, 36) | 30 (25, 36) | 31 (26, 35) | 0.304 |
| **Comorbidities, n(%)** |  |  |  |  |
| MI, n (%) | 95 (9) | 57 (8) | 38 (11) | 0.122 |
| CHF, n (%) | 157 (15) | 84 (12) | 73 (22) | < 0.001 |
| Renal disease, n (%) | 128 (12) | 58 (8) | 70 (21) | < 0.001 |
| RD, n (%) | 16 (2) | 14 (2) | 2 (1) | 0.149 |
| PVD, n (%) | 70 (7) | 46 (7) | 24 (7) | 0.827 |
| CPD, n (%) | 120 (12) | 72 (10) | 48 (14) | 0.073 |
| Hypertension, n (%) | 275 (27) | 172 (25) | 103 (31) | 0.043 |
| **Vital signs** |  |  |  |  |
| Mean HR (min^-1^ ) | 78.2 (70.06, 87.87) | 77.71 (69.57, 87.6) | 79.38 (71.06, 87.89) | 0.199 |
| Mean AP (mmHg) | 85.83 ± 10.03 | 86.16 ± 10.1 | 85.14 ± 9.87 | 0.127 |
| Mean RR (min^-1^) | 18.35 (16.79, 20.21) | 18.34 (16.81, 20.2) | 18.36 (16.76, 20.28) | 0.904 |
| Mean Temp (°C) | 36.94 (36.73, 37.24) | 36.94 (36.73, 37.23) | 36.94 (36.74, 37.26) | 0.872 |
| Mean SpO2 (%) | 97.08 (95.92, 98.34) | 97.14 (95.93, 98.3) | 97.06 (95.92, 98.48) | 0.963 |
| **Laboratory test** |  |  |  |  |
| WBC (K/uL) | 9.95 (7.93, 12.78) | 10.15 (8, 12.9) | 9.7 (7.5, 12.4) | 0.041 |
| Neutrophils (%) | 79.9 (70.35, 85.8) | 80.3 (71.5, 86) | 78.3 (68.7, 84.8) | 0.072 |
| Lymphocytes (%) | 25 (15.8, 38.8) | 24.4 (16, 36.95) | 27 (15, 41.3) | 0.141 |
| Hemoglobin (g/dL) | 12.7 (11.45, 13.8) | 12.6 (11.45, 13.65) | 12.8 (11.45, 14.15) | 0.044 |
| Platelets (K/uL) | 209 (171, 257.88) | 211.25 (173.12, 257.38) | 203.75 (166.75, 258.12) | 0.412 |
| RDW (%) | 13.6 (13, 14.49) | 13.6 (13.05, 14.5) | 13.6 (12.89, 14.4) | 0.103 |
| Calcium (mg/dL) | 8.8 (8.4, 9.2) | 8.8 (8.4, 9.15) | 8.9 (8.51, 9.25) | 0.130 |
| Sodium (mEq/L) | 139.5 (137.5, 142) | 139.5 (137.5, 142) | 140 (137.5, 142) | 0.518 |
| Potassium (mEq/L) | 4 (3.7, 4.3) | 4 (3.75, 4.35) | 4 (3.7, 4.25) | 0.252 |
| Creatinine (mg/dL) | 0.9 (0.75, 1.1) | 0.9 (0.7, 1.1) | 0.9 (0.75, 1.11) | 0.109 |
| BUN, Median (mg/dL) | 16.5 (13, 21.5) | 16.5 (13, 21.5) | 17 (12.5, 21.5) | 0.972 |
| Bilirubin (mg/dL) | 0.6 (0.4, 0.9) | 0.6 (0.4, 0.9) | 0.65 (0.4, 0.9) | 0.254 |
| ALT **^b^** | 1.28 (1.18, 1.45) | 1.28 (1.15, 1.45) | 1.3 (1.18, 1.45) | 0.308 |
| AST **^c^** | 1.41 (1.3, 1.58) | 1.41 (1.3, 1.57) | 1.41 (1.3, 1.59) | 0.744 |
| PT (s) | 12.4 (11.5, 13.8) | 12.4 (11.53, 13.8) | 12.4 (11.5, 13.62) | 0.733 |
| APTT (s) | 27.95 (25.45, 30.85) | 27.8 (25.4, 30.9) | 28.12 (25.79, 30.61) | 0.519 |
| Triglycerides (mg/dL) | 94 (71, 138) | 96 (72, 137.5) | 93 (69, 136.75) | 0.596 |
| ABG (mg/dL) | 124 (103, 155) | 115 (99, 135) | 164 (130, 216) | < 0.001 |
| HbA1C (%) | 5.7 (5.4, 6.3) | 5.5 (5.2, 5.8) | 6.9 (6.3, 8.0) | < 0.001 |
| **Events** |  |  |  |  |
| Los ICU (days) | 3.32 (1.73, 7.59) | 3.27 (1.75, 7.23) | 3.39 (1.71, 8.02) | 0.409 |
| Los Hospital (days) | 7.63 (4.05, 14.82) | 7.57 (4.02, 14.13) | 7.9 (4.43, 15.63) | 0.582 |
| 30-day mortality (%) | 195 (19) | 134 (19) | 61 (18) | 0.777 |
| 1-year mortality (%) | 323 (31) | 215 (31) | 108 (32) | 0.680 |
| Lung infection (%) | 168 (16) | 108 (16) | 60 (18) | 0.360 |
| **Medications** |  |  |  |  |
| Vasoactive drugs | 148 (14) | 98 (14) | 50 (15) | 0.894 |
| RRT | 20 (2) | 13 (2) | 7 (2) | 0.772 |
| Invasive ventilation | 384 (37) | 273 (39) | 111 (33) | 0.089 |
| Vent time (hours) | 40.34 (22.5, 108.87) | 40.34 (22, 110.5) | 39.38 (23.36, 103.35) | 0.876 |

*^a^ Continuous data is presented as median (interquartile range), whereas categorical data is presented as frequency (percentage)*

***^b^*** *ALT in the table is the value after logarithmic transformation.*

***^c^*** *AST in the table is the value after logarithmic transformation.*

*Abbreviation: SHR, stress hyperglycemia ratio; GCS, Glasgow coma scale; APSIII, acute physiology score III; SAPSII, simplifed acute physiological score II; SOFA, sequential organ failure assessment; OASIS, oxford acute severity of illness score;* *MI, myocardial infarct; CHF,* *congestive heart failure; RD,* *rheumatic disease; PVD, peripheral vascular disease; CPD, chronic pulmonary disease; HR: heart rate; AP: arterial pressure; RR: respiratory rate; Temp: body temperature; WBC, white blood cell; RDW, red cell distribution width; PT, prothrombin time; APTT, activated partial thromboplastin time; ABG：admission blood glucose;* HbA1C: glycated hemoglobin A1; RRT, renal replacement treatment.
