# Supplementary material for: Prognostic significance of the stress hyperglycemia ratio and admission blood glucose in diabetic and nondiabetic patients with spontaneous intracerebral hemorrhage
Source: Diabetol Metab Syndr. 2024 Mar 4;16:58. doi: 10.1186/s13098-024-01293-0 (PMC10910766; doi:10.1186/s13098-024-01293-0)
Supplement: Supplementary file 5 — Supplementary Material 5 [file 13098_2024_1293_MOESM5_ESM.docx]

**Table S3. Hazard ratio (HR) for all-cause 30-day or 1-year mortality of SHR and ABG in the patients without diabetes**

| **Variables** | **30-day mortality** | | | | | | |  | **1-year mortality** | | | | | | |
| --- | --- | --- | --- | --- | --- | --- | --- | --- | --- | --- | --- | --- | --- | --- | --- |
|  | **Unadjusted model** | | |  | **Adjusted model ^d^** | | |  | **Unadjusted model** | | |  | **Adjusted model ^e^** | | |
|  | **HR（95% CI)** | ***p value*** | ***p-trend*** |  | **HR（95% CI)** | ***p value*** | ***p-trend*** |  | **HR（95% CI)** | ***p value*** | ***p-trend*** |  | **HR（95% CI)** | ***p value*** | ***p-trend*** |
| **SHR** |  |  |  |  |  |  |  |  |  |  |  |  |  |  |  |
| Continuous variable  per unit | 3.76 (2.80,5.04) | <0.001 |  |  | 3.24 (2.11,4.98) | <0.001 |  |  | 3.06 (2.34,4.01) | <0.001 |  |  | 2.31 (1.64,3.25) | <0.001 |  |
| Quantile **^a^** |  |  | <0.001 |  |  |  | 0.003 |  |  |  | <0.001 |  |  |  | <0.001 |
| Q1 (n=145) | Ref |  |  |  |  |  |  |  | Ref |  |  |  |  |  |  |
| Q2 (n=144) | 1.35 (0.65,2.77) | 0.420 |  |  | 1.47 (0.69,3.13) | 0.317 |  |  | 1.13 (0.70,1.83) | 0.611 |  |  | 1.22 (0.75,2.00) | 0.428 |  |
| Q3 (n=145) | 1.92 (0.98,3.78) | 0.056 |  |  | 2.06 (1.01,4.19) | 0.047 |  |  | 1.32 (0.83,2.10) | 0.243 |  |  | 1.52 (0.93,2.47) | 0.092 |  |
| Q4 (n=145) | 2.79 (1.47,5.30) | 0.002 |  |  | 2.92 (1.46,5.84) | 0.003 |  |  | 1.66 (1.07,2.60) | 0.027 |  |  | 2.02 (1.24,3.28) | 0.005 |  |
| Q5 (n=145) | 5.12 (2.79,9.38) | <0.001 |  |  | 4.55 (2.33,8.88) | <0.001 |  |  | 2.99 (1.97,4.54) | <0.001 |  |  | 3.06 (1.93,4.86) | <0.001 |  |
| **ABG** |  |  |  |  |  |  |  |  |  |  |  |  |  |  |  |
| Continuous variable  per unit | 1.01 (1.00,1.01) | <0.001 |  |  | 1.01 (1.00,1.02) | <0.001 |  |  | 1.01 (1.00,1.01) | <0.001 |  |  | 1.01 (1.00,1.01) | <0.001 |  |
| Quantile **^b^** |  |  | <0.001 |  |  |  | <0.001 |  |  |  | <0.001 |  |  |  | <0.001 |
| Q1 (n=145) | Ref |  |  |  |  |  |  |  | Ref |  |  |  |  |  |  |
| Q2 (n=150) | 0.47 (0.22,1.00) | 0.049 |  |  | 0.53 (0.24,1.16) | 0.111 |  |  | 0.66 (0.41,1.08) | 0.096 |  |  | 0.84 (0.51,1.38) | 0.486 |  |
| Q3 (n=148) | 1.54 (0.87,2.71) | 0.136 |  |  | 1.81 (0.99,3.30) | 0.055 |  |  | 1.23 (0.80,1.88) | 0.346 |  |  | 1.55 (0.98,2.45) | 0.060 |  |
| Q4 (n=140) | 1.54 (0.87,2.73) | 0.143 |  |  | 1.66 (0.90,3.07) | 0.107 |  |  | 1.13 (0.73,1.75) | 0.592 |  |  | 1.38 (0.86,2.23) | 0.181 |  |
| Q5 (n=141) | 3.32 (1.98,5.56) | <0.001 |  |  | 2.71 (1.55,4.73) | <0.001 |  |  | 2.54 (1.72,3.74) | <0.001 |  |  | 2.38 (1.56,3.63) | <0.001 |  |

***^a^*** *SHR: Q1 (≤0.873), Q2 (0.873-0.978), Q3 (0.978-1.100), Q4 (1.100-1.260) , Q5 (＞1.260).*

***^b^*** *Glucose: Q1 (≤97.6), Q2 (97.6-110), Q3 (110-123), Q4 (123-144) , Q5 (＞144).*

***^c^****Adjusted model was adjusted for the variables with a p value<0.01 in the univariable Cox regression, including age, heart rate, respiratory rate, body temperature, SpO_2,_ Glasgow Coma Scale, white blood cell count, lymphocyte percentage, hemoglobin, platelets, red blood cell distribution width, serum calcium, serum sodium, triglycerides, creatinine, blood urea nitrogen, aspartate aminotransferase, and bilirubin.*

***^d^*** *Adjusted model was adjusted for the variables with a p value<0.01 in the univariable Cox regression, including gender, age, myocardial infarct, congestive heart failure, renal disease, heart rate, mean arterial pressure, respiratory rate, SpO_2_, Glasgow Coma Scale, white blood cell count, hemoglobin, red cell distribution width, serum sodium, creatinine, and blood urea nitrogen.*
