# Supplementary material for: Prognostic significance of the stress hyperglycemia ratio and admission blood glucose in diabetic and nondiabetic patients with spontaneous intracerebral hemorrhage
Source: Diabetol Metab Syndr. 2024 Mar 4;16:58. doi: 10.1186/s13098-024-01293-0 (PMC10910766; doi:10.1186/s13098-024-01293-0)
Supplement: Supplementary file 6 — Supplementary Material 6 [file 13098_2024_1293_MOESM6_ESM.docx]

**Table S4. Discrimination ability of SHR and ABG for all cause 30-day and 1-year mortality in patients without diabetes.**

| **Variables** | **30-day mortality** | |  | **1-year mortality** | |
| --- | --- | --- | --- | --- | --- |
|  | **C-Statistic** | ***p value*** |  | **C-Statistic** | ***p value*** |
| APSIII | 0.712 | Ref |  | 0.683 | Ref |
| APSIII+SHR | 0.745 | <0.001 |  | 0.703 | 0.002 |
| APSIII+ABG | 0.743 | <0.001 |  | 0.705 | <0.001 |
|  |  |  |  |  |  |
| SAPSII | 0.741 | Ref |  | 0.729 | Ref |
| SAPSII+SHR | 0.776 | <0.001 |  | 0.749 | <0.001 |
| SAPSII+ABG | 0.768 | <0.001 |  | 0.748 | <0.001 |
|  |  |  |  |  |  |
| SOFA | 0.684 | Ref |  | 0.660 | Ref |
| SOFA+SHR | 0.739 | <0.001 |  | 0.696 | 0.002 |
| SOFA+ABG | 0.732 | <0.001 |  | 0.694 | <0.001 |
|  |  |  |  |  |  |
| OASIS | 0.707 | Ref |  | 0.681 | Ref |
| OASIS+SHR | 0.750 | <0.001 |  | 0.707 | <0.001 |
| OASIS+ABG | 0.737 | <0.001 |  | 0.701 | <0.001 |

*SHR, stress hyperglycemia ratio; ABG, admission plasma glucose; APSIII, acute physiology score III; SAPSII, simplified acute physiological score II; SOFA, sequential organ failure assessment; OASIS, oxford acute severity of illness score; SHR, stress hyperglycemia ratio.*
