# Supplementary material for: Prognostic significance of the stress hyperglycemia ratio and admission blood glucose in diabetic and nondiabetic patients with spontaneous intracerebral hemorrhage
Source: Diabetol Metab Syndr. 2024 Mar 4;16:58. doi: 10.1186/s13098-024-01293-0 (PMC10910766; doi:10.1186/s13098-024-01293-0)
Supplement: Supplementary file 7 — Supplementary Material 7 [file 13098_2024_1293_MOESM7_ESM.docx]

**Table S5. Discrimination ability of SHR and ABG for all cause 30-day and 1-year mortality in patients with diabetes.**

|  | **30-day mortality** | |  | **1-year mortality** | |
| --- | --- | --- | --- | --- | --- |
|  | **C-Statistic** | ***p value*** |  | **C-Statistic** | ***p value*** |
| APSIII | 0.615 | Ref |  | 0.626 | Ref |
| APSIII+SHR | 0.663 | 0.003 |  | 0.644 | 0.052 |
| APSIII+ABG | 0.639 | 0.084 |  | 0.629 | 0.504 |
|  |  |  |  |  |  |
| SAPSII | 0.695 | Ref |  | 0.714 | Ref |
| SAPSII+SHR | 0.726 | 0.004 |  | 0.719 | 0.066 |
| SAPSII+ABG | 0.706 | 0.073 |  | 0.714 | 0.282 |
|  |  |  |  |  |  |
| SOFA | 0.642 | Ref |  | 0.660 | Ref |
| SOFA+SHR | 0.692 | <0.001 |  | 0.667 | 0.017 |
| SOFA+ABG | 0.656 | 0.124 |  | 0.660 | 0.180 |
|  |  |  |  |  |  |
| OASIS | 0.692 | Ref |  | 0.677 | Ref |
| OASIS+SHR | 0.713 | 0.003 |  | 0.682 | 0.036 |
| OASIS+ABG | 0.698 | 0.231 |  | 0.674 | 0.191 |

*SHR, stress hyperglycemia ratio; ABG, admission plasma glucose; APSIII, acute physiology score III; SAPSII, simplified acute physiological score II; SOFA, sequential organ failure assessment; OASIS, oxford acute severity of illness score; SHR, stress hyperglycemia ratio; ABG, admission plasma glucose.*
