# Supplementary material for: Prognostic significance of the stress hyperglycemia ratio and admission blood glucose in diabetic and nondiabetic patients with spontaneous intracerebral hemorrhage
Source: Diabetol Metab Syndr. 2024 Mar 4;16:58. doi: 10.1186/s13098-024-01293-0 (PMC10910766; doi:10.1186/s13098-024-01293-0)
Supplement: Supplementary file 9 — Supplementary Material 9 [file 13098_2024_1293_MOESM9_ESM.docx]

**Table S7. Comparison of the predictive efficiency between SHR and ABG for all cause 30-day and 1-year mortality in patients with diabetes.**

| **Model 1** | **C-Statastic** | **Model 2** | **C-Statastic** | ***p value ^a^*** |
| --- | --- | --- | --- | --- |
| **30-day mortality** |  |  |  |  |
| SHR | 0.622 | ABG | 0.588 | <0.001 |
| APSIII+SHR | 0.663 | APSIII+ABG | 0.639 | <0.001 |
| SAPSII+SHR | 0.726 | SAPSII+ABG | 0.706 | <0.001 |
| SOFA+SHR | 0.692 | SOFA+ABG | 0.656 | <0.001 |
| OASIS+SHR | 0.713 | OASIS+ABG | 0.698 | <0.001 |
|  |  |  |  |  |
| **1-year mortality** |  |  |  |  |
| SHR | 0.564 | ABG | 0.545 | <0.001 |
| APSIII+SHR | 0.644 | APSIII+ABG | 0.629 | <0.001 |
| SAPSII+SHR | 0.719 | SAPSII+ABG | 0.714 | <0.001 |
| SOFA+SHR | 0.667 | SOFA+ABG | 0.660 | <0.001 |
| OASIS+SHR | 0.682 | OASIS+ABG | 0.674 | <0.001 |

**^a^***p-value is the result of the C-statistics comparison between model 1 and model 2 on the same row.*

*SHR, stress hyperglycemia ratio; ABG, admission plasma glucose; APSIII, acute physiology score III; SAPSII, simplified acute physiological score II; SOFA, sequential organ failure assessment; OASIS, oxford acute severity of illness score; SHR, stress hyperglycemia ratio.*
